# Nutritional Assessment, Body Composition, and Low Energy Availability in Sport Climbing Athletes of Different Genders and Categories: A Cross-Sectional Study

**DOI:** 10.3390/nu16172974

**Published:** 2024-09-03

**Authors:** Agustin Mora-Fernandez, Andrea Argüello-Arbe, Andrea Tojeiro-Iglesias, Jose Antonio Latorre, Javier Conde-Pipó, Miguel Mariscal-Arcas

**Affiliations:** 1 Health Science and Nutrition Research (HSNR-CTS1118), Department of Nutrition and Food Science, School of Pharmacy, University of Granada, 18071 Granada, Spain; agusmora@correo.ugr.es (A.M.-F.); nutricion.arguello@gmail.com (A.A.-A.); toandre@live.com (A.T.-I.); javiercondepipo@gmail.com (J.C.-P.); 2 Department of Food Technology, Nutrition and Food Science, Campus of Lorca, University of Murcia, 30800 Murcia, Spain; joseantonio.latorre@um.es; 3 Instituto de Investigación Biosanitaria de Granada (ibs.GRANADA), 18012 Granada, Spain

**Keywords:** nutrition assessment, sport climbing, bouldering, rock climbing, low energy availability, dietary intake, relative energy deficiency in sport

## Abstract

Climbing is an Olympic discipline in full development and multidisciplinary in nature, where the influences of body composition and nutritional status on performance have not yet been clarified despite the quest for a low weight in anti-gravity disciplines such as climbing. The present cross-sectional study aimed to conduct nutritional (3-day dietary diaries) and body composition (ISAK profile) assessments on sport climbing athletes by gender and climbing level during the months of February and March 2024. The *t*-test for independent samples and the Mann–Whitney U-test, as well as an ANOVA and the Kruskal–Wallis H-test, were used to compare the distributions of two or more groups, respectively, and Pearson’s and Spearman’s correlation coefficients were used to estimate the correlations between the different variables. The mean age of the 46 Spanish climbers (22 men and 24 women) was 30 years (SD: 9) with 7.66 years of experience (SD: 6.63). The mean somatotype of the athletes was classified as balanced mesomorph. Negative correlations were observed between fat mass variables and climbing level (*p* < 0.010), and positive correlations were observed with forearm circumference (*p* < 0.050). The mean energy availability (EA) was 33.01 kcal-kg FFM^−1^d^−1^ (SD: 9.02), with 55.6% of athletes having a suboptimal EA status and 35.6% having low energy availability (LEA). The carbohydrate and protein intakes were below the recommendations in 57.8% and 31.1% of athletes, respectively. There were deficient intakes of all micronutrients except phosphorus in males. These findings suggest that climbing athletes are at a high risk of developing low energy availability states and concomitant problems. Optimal nutritional monitoring may be advisable in this type of athlete to try to reduce the risk of LEA.

## 1. Introduction

Sport climbing is a relatively young discipline and highly diverse in its practise given the wide variety of environments or permutations of routes, among many others, where categorisation at the individual level is particularly complex [1]. Since the first World Championships held in Germany in 1991, sport climbing has experienced a growing competitive development, even becoming part of the official programme of the Tokyo Summer Olympic Games in 2020 with three disciplines of differential character, such as speed, lead, and bouldering (with the latter two being called difficulty disciplines) [2]; it has become a sport with a huge increase in the number of events and practitioners at the international level, with more than 20 million people around the world being practitioners of some of its disciplines [2,3].

In sport climbing, participants are required to conquer climbing routes, either on natural rock or artificial walls, in real-time competitions, encompassing both indoor and outdoor environments [2]. In lead climbing, for example, typical ascent times range from 2 to 7 min, and oxygen consumption (VO_2_) averages around 20–25 mL-kg^1^-min^1^ during this period [4]. This activity is characterised by a predominant contribution of the aerobic and alactic anaerobic systems, with the economy of effort having an important role [5].

Despite its competitive nature, the factors that influence climbing performance do not seem to be well understood to date, suggesting that it is a multifactorial sport discipline where performance seems to be influenced by numerous factors [3,6,7,8].

However, although body composition is not shown to be a major determinant of climbing athlete performance [9,10,11,12], the tendency towards relatively low levels of body weight and body fat appears to be a common pattern in this type of athlete [9,11]. The potential effects of body lightness on climbing [12] mean that the anti-gravitational nature of this sport poses an added risk for these athletes to suffer from states of low energy availability (LEA) and specific nutritional imbalances [13,14].

These long-term energy deficiencies can lead to adverse physiological and psychological outcomes as well as possible unfavourable effects on the health and athletic performance of these athletes [15], conceptualised in what is now known as the Female Athlete Triad, the Male Athlete Triad, and the Relative Energy Deficiency in Sport (RED-S) models [16]. Examples of these adverse health effects in RED-S athletes include impairments in bone health; impairments in reproductive, neurocognitive, gastrointestinal, cardiovascular, and metabolic functions; as well as reductions in immunity and musculoskeletal function, among many others [15].

The anthropometric differences between sport climbing athletes with respect to the general population [12], as well as the practise of a greater number of climbing athletes with increased tendencies towards energy imbalances, such as the female athlete population [16], highlight the need for an adequate nutritional status in these athletes with the aim of preventing RED situations in this type of vulnerable group [17,18].

These differences are also reflected in the proposal of different specific nutritional recommendations adjusted to the practise of sport climbing, particularly for the female climbing athlete [13,14,19], to try to alleviate nutritional deficiencies and optimise the state of health and performance of these athletes. Although previous studies have already reported a compromised nutritional status with relatively low energy and carbohydrate intakes in international climbing athletes [13,14], the situation in the Spanish climbing athletes of each gender and ability level is totally unknown. Taking into account these precedents and the added nutritional risk presented by sport climbing athletes, it is possible to expect an influence of this sport practise on the body composition, nutritional status, and energy availability of this population group in Spain. Also, considering the close link between the athlete’s nutritional habits and their health status and sports [20], it becomes a fundamental aspect to determine the current nutritional overview in climbing athletes of different genders and levels in order to detect groups that are more compromised. Therefore, this study sought to evaluate body composition and nutritional status in Spanish climbing athletes.

## 2. Materials and Methods

### 2.1. Design

A descriptive, observational, and cross-sectional study design was used, where anthropometric and body composition assessments were performed on the subjects, and dietary diaries were completed [21].

All of these tests were conducted in person in the period between February and March 2024 during camps and athlete concentrations at the climbing centre called Sputnik Climbing (Las Rozas, Madrid, Spain) and the climbing gym called GekoAventura (Valladolid, Spain). The participants belonged to the professional climbing team named Sputnik Climbing (Las Rozas, Madrid, Spain) or were members of the aforementioned climbing centres. The latter were recruited voluntarily in different calls for applications through posters and publications in social networks made in the climbing centres, and they were informed about the purpose and requirements of this study. Athletes who practised sport climbing in any modality and conditions on a regular basis (weekly climbing practise), aged between 16 and 50 years, with no pathologies or use of drugs that could interfere with the study, and with a minimum of 1 year of experience were included. Pregnant athletes were excluded from the study.

Data collection was carried out during a single visit per subject, with the subject being shown the corresponding instructions to enable them to complete the dietary records. They were provided with the contact details of two researchers (AMF and AAA) for the submission of the dietary records.

### 2.2. Ethical Issues

This study complied with the principles of the European Code of Conduct for Research Integrity [22] and followed the steps of the EU General Data Protection Regulation 2016/679 (GDPR) [23]. This study was approved by the Ethics Committee of the University of Granada (3340/CEIH/2023).

Prior to the start of this study, all participants and/or their legal guardians were required to complete an informed consent form regarding the use and protection of the data to be included in the study.

### 2.3. Sport Characteristics

Participants were surveyed according to the recommended standards of the IRCRA (International Rock Climbing Research Association) position statement to improve the comparability of this study [24]. Therefore, they answered a series of questions on predominant discipline, percentages of time spent in the past 3/12 months in each discipline and indoor/outdoor climbing, weekly “net” climbing hours in the past 3 and 12 months, years of experience, and participation/presence in national competitions. In addition, the highest consolidated degree of climbing difficulty (a wall or surface successfully climbed repeatedly) by each of the participants in the past month was subjectively reported according to the International Rock Climbing Research Association reporting scale [24] to facilitate a common approach to the data.

### 2.4. Body Composition

Body weights were measured with a Tanita BC-545N electronic scale (TANITA Corp. Tokio, Japan). These measurements were taken on an empty stomach, first thing in the morning, with the subjects undressed.

For height, a SECA (SECA, Hamburg, Germany) wall-mounted measuring tape with a range of 220 cm and a 1 mm division was used, as well as an anthropometric box 50 cm high and 40 cm wide to measure sitting height. Both measurements were taken during inspiration with the subjects positioned in the Frankfort plane, and the height of the anthropometric box was then subtracted from the seated height. Similarly, the arm span was taken at the end of inspiration with the subject resting the dactylion point of the right hand on the end (corner) of the wall, and the dactylion point of the left hand was marked with a pen on the wall itself, trying to reach the maximum distance between the two points [25].

The rest of the anthropometric data were obtained as follows: a Harpenden Holtainpicometre (Crosswell, Crymych, Pembs., SA41 3UF, UK, accuracy of 0.2 mm and range of 0–80 mm) was used for the measurement of skinfolds (triceps, subscapular, biceps, iliac crest, supraspinal, abdominal, thigh, and calf); sliding calliper (resolution of 1 mm and measurement range of 1–20 cm) for the measurement of bone diameters (humerus, bi-styloid, and femur); Cercorf segmometer (Equipamentos Antropometricos, Av. Copacabana, 435—Tristeza—Porto Alegre/RS, Brazil, accuracy of 0.5 mm and measuring range of 3 m) for hand length measurement (midstylion-dactylion); and a Cescorf tape measure (Equipamentos Antropometricos, Av. Copacabana, 435—Tristeza—Porto Alegre/RS, Brazil, accuracy of 1 mm, range of 2 m) for girth measurement (arm relaxed, arm flexed, forearm, thigh middle, and calf) and parallel to the ground for wingspan.

Anthropometric assessment of body composition was performed using the International Society for the Advancement of Kineanthropometry (ISAK) restricted profile by an ISAK level 2 certified practitioner [25,26]. Two measurements were taken for each measurement, with the mean value reported if the technical error of measurement was <5%. If the technical error of measurement was >5%, a third measurement was taken, and the median of the 3 values was reported.

The sum of 6 skinfolds (triceps, subscapular, suprascapular, supraspinal, abdominal, thigh, and calf) and 8 skinfolds (triceps, subscapular, biceps, iliac crest, supraspinal, abdominal, thigh, and calf) were estimated for their application in sports nutrition and association with the individual adiposity index of each athlete [27,28].

Given its applicability and relationship with dual-energy X-ray absorptiometry, the Slaughter–Lohman and Poortmans formulas were used to estimate the weight in kilograms of fat mass and muscle mass, respectively, in participants aged 8–18 years [29], and the Durnin and Womersley (fat mass in kg) and Lee (muscle mass in kg) formulas were used for the rest of the participants [30,31]. Rocha’s formula was used to estimate bone mass of all participants [32].

Percentage values of fat mass were estimated using Durnin and Womersley formula, taking into account their application in field work and the lower estimation bias compared to other approaches shown in previous studies with climbing athletes with respect to dual-energy X-ray absorptiometry (DXA) [30,33].

Once the anthropometric measurements were collected, the different components of the somatotype were calculated from the method used by Health and Carter [34] using the following equations that were used in previous work with climbers [35]:

Endomorphy: −0.7182 + 0.1451(x) − 0.00068(x2) + 0.0000014(x3), where x is the sum of the triceps, subscapular, and supraspinal folds multiplied by 70.18/height in cm.

Mesomorphy: (0.858 × H) + (0.601 × F) + (0.188 × B) + (0.161 × P) − (0.131 × E) + 4.5, where H is the biepicondilar breadth of the humerus, F is the bicondilar breadth of the femur, B is the flexed arm circumference, P is the perimeter of the calf, and E is the height.

Ectomorphy: For this parameter, there are three formulas based on the weight index, which were the results of the following formula: WI = Height a√Weight.

If WI ≥ 40.75, the formula was 0.732 × WI − 28.58;

If WI < 40.75 but >38.25, the formula was 0.463 × WI − 17.63;

If WI ≤ 38.25, the given value was 0.1.

### 2.5. Nutritional or Dietary Assessment

Athletes were subjected to prospective measurements (dietary diary) of dietary intake as a gold standard for an adequate nutritional or dietary assessment [36].

#### 2.5.1. Dietary Diary and Adjustment to Requirements

Participants were instructed by two experienced members of the research team (AM-F and AA-A) to weigh (in grams) or measure (in millilitres) all foods, fluids, and dietary supplements consumed in a 3-day non-consecutive training period to determine the dietary diary, which is an accepted method used in previous research to record dietary intake in sport climbing athletes [21,36]. Weightings were preferably performed using proprietary digital kitchen scales or by reporting the manufacturer’s weights and quantities, using alternative standardised home measures reported by the research team (250 mL cups, tablespoons, and dessert spoons, for example) in exceptional situations. Participants were also provided with a manual with notes and guidelines on accurate reporting requirements. For the registration of precooked or packaged products, as well as for the registration of dietary supplements, participants were asked to report the brand, weight of the product, and nutritional values, specifying the dosage and type of supplement used specifically.

Subsequently, the records were analysed using the nutritional software based on the cloud computing technology DietoPro.com [37]. The estimated energy intakes of the athletes were compared with the estimates made in the previous section to calculate energy availability. On the one hand, the macronutrient intakes (carbohydrates, proteins, and fats) of the athletes in the sample were compared with the nutritional recommendations proposed by recent review articles for rock climbing athletes, using ranges of 3–7 g CHO/kg per day and an intake of 20–35% of the total energy content from fat as reference [13,14]. The reference protein intakes were adjusted according to the predominant discipline of the athlete, using values of 1.4–2 g/kg BM in boulderers and 1.2–1.8 g/kg BM for other climbing athletes as references [14]. Moreover, the estimated average intakes of vitamins (thiamine, riboflavin, niacin, folic acid, and vitamins B6, B12, C, A, D, and E) and minerals (Ca, Fe, P, K, Mg, Zn, I, and Se) for each participant were compared with the Dietary Reference Intakes (DRI) for the Spanish population established by the Spanish Federation of Nutrition, Food and Dietetics Societies (FESNAD) in 2010 [38].

#### 2.5.2. Estimation of Energy Availability

To assess the energy availability (EA) of each athlete, exercise energy expenditure (EEE) was calculated from the “net” climbing time (time per week spent climbing surfaces of any type) declared in the sports characteristics questionnaire and its conversion to kilocalories using the metabolic equivalent of the task (MET) corresponding to the values for climbing included in the Compendium of Physical Activities [39,40]. For the calculation of fat-free mass (FFM), the subject’s body weight was subtracted from the values obtained for FM from the Slaughter–Lohman and Durnin and Womersley formulas based on the anthropometric analysis of each athlete.

The EA in relation to the fat-free mass of the sample was finally estimated from the values of EEE, energy intake, and FFM of each athlete using the following formula [41,42]:

EA = (EI − EEE)/FFM

Athletes were classified as LEA with values < 30 kcal-kg FFM1-d1, suboptimal EA with values < 45 kcal-kg FFM1-d1, and optimal EA with values > 45 kcal-kg FFM1-d1 [43,44].

### 2.6. Statistical Analysis

Sample size calculation was performed using G* POWER software v.3.1.9.7 (Heinrich-Heine-Universität Düsseldorf, Düsseldorf, Germany) [45]. Taking into account a dropout rate of less than 10% with an alpha of 0.05, an effect size of 0.75, and a statistical power of 0.95, a total sample size of 46 subjects was required for the comparison of a total of 6 groups, with a minimum sample size of 7 athletes required for each group.

Statistical analysis was carried out using SPSS Statistics software (version 27, IBM New York, NY, USA). Means, standard deviations, frequencies, and percentages were used for basic description. Normality was calculated using the Shapiro–Wilk test. Homogeneity of variances was estimated using Levene’s test. For comparison of independent group distributions, the independent samples *t*-test was used for normally distributed data. The non-parametric Mann–Whitney test was used when the normality of the distribution was not respected. Similarly, to analyse the differences between more than 2 independent groups in normal variables, the ANOVA test was used, while if the distribution of the variables did not follow a normal distribution, the nonparametric Kruskal–Wallis H test was used. The Tukey and Games–Howell post hoc tests and the Dunn test were used to perform the analysis of the differences by subgroups in the ANOVA and Kruskal–Wallis tests, respectively [46]. Pearson’s correlation coefficient (R) was used to correlate those variables with a normal distribution. Spearman’s correlation coefficient was used when the normality of the distribution was not respected. Correlation values (R) were set as follows: <0.2: weak correlation, 0.2–0.8: medium correlation, and >0.8: strong correlation [21]. The magnitude of the difference in effect size was obtained with Cohen’s d index, which was interpreted as null (0–0.19), small (0.20–0.49), medium (0.50–0.7), and large (≥0.80) [47]. The level of statistical significance for all tests was set at 95%.

## 3. Results

### 3.1. Sample Characteristics

The demographic data and sporting characteristics of the participants are shown in Table 1. A total of 46 climbers (22 men and 24 women with 7.66 years (SD: 6.63) of experience) from Spain, aged between 17 and 50 years (mean age 30 years (SD: 9)), participated in the present study. According to the IRCRA classification, a total of 14 participants (7 males and 7 females) were classified as intermediate (level 2), 18 (8 males and 10 females) as advanced (level 3), 11 (6 males and 5 females) as elite (level 4), and 3 (1 male and 2 females) as higher elite (level 5), although the latter were integrated inside the “elite” athletes group. A total of 50% of athletes reported bouldering as the predominant discipline, while 2.2% and 47.8% reported speed and lead, respectively. Of the 46 subjects, 11 (6 males and 5 females) reported current or past participation in state or international championships, while the remaining 35 did not report having participated in any events.

Statistically significant differences with large effect sizes were only observed for weight (*p* < 0.010, d = 2.487, and CI = 1.703–3.256), height (*p* < 0.010, d = 2.284, and CI = 1.527–3.026), and BMI (*p* < 0.010, d = 1.409, and CI = 0.754–2.051) between the two genders.

### 3.2. Body Composition

**Figure 1:**
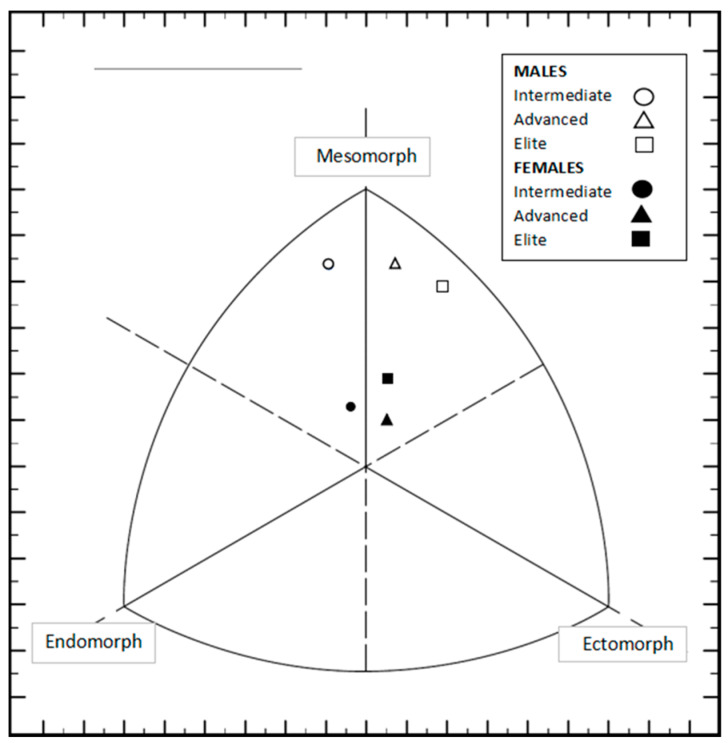
Mean somatotype values for different groups of athletes.

The values of the different body composition variables according to the different classifications or climbing levels are shown in Table 2. The somatotype of the male climbers was classified with values typical of an ecto-mesomorphic somatotype (endomorphy: 2.17 (SD: 0.90); mesomorphy: 5.95 (SD: 0.77); ectomorphy: 2.81 (SD: 0.84)), while that of the female athletes fell within a balanced mesomorphic somatotype (endomorphy: 2.82 (SD: 0.57); mesomorphy: 4.04 (SD: 0.85); ectomorphy: 3.14 (SD: 0.86)), with the total somatotype of the sample being classified as a balanced mesomorphic type (endomorphy: 2.51 (SD: 0.81); mesomorphy: 4.95 (SD: 1.26); ectomorphy: 2.98 (SD: 0.86)). The mean somatotype values for the different groups of athletes are represented in Figure 1.

Significant differences were observed between the intermediate and elite groups in the values of the sum of the six and eight skinfolds (*p* < 0.010), kilograms of fat mass (*p* < 0.010), endomorphy (*p* < 0.010), and ectomorphy (*p* < 0.050), while significant differences between the advanced–intermediate and advanced–elite groups were shown in the values of ectomorphy (*p* < 0.050), mid-thigh circumference (*p* < 0.050), and kg of fat mass (*p* < 0.050), respectively.

Statistically highly significant (*p* < 0.010) medium negative correlations were observed between all variables related to the athletes’ body fat and IRCRA scores. Medium correlations were found between endomorphy (r = −0.589), ∑6 (r = −0.573), ∑8 (r = −0.616), % of fat mass (r = −0.497), and fat mass in kilograms (r = −0.590) with respect to the athletes’ IRCRA scores.

Statistically significant (*p* < 0.050) moderate positive correlations were also observed between the IRCRA scores of the sample with respect to the values of ectomorphy (r = 0.308) and forearm girth (r = 0.342). No significant correlations were found between the remaining body composition variables and the IRCRA scores of the sample.

### 3.3. Dietary Intake

Despite instructions and tools being provided to each participant, one female intermediate athlete (IRCRA scale: 14) did not report her dietary intake from the 3-day dietary diary, resulting in a final dropout rate of 2.2%, and the dietary data provided by the remaining 45 athletes are included below.

#### 3.3.1. Energy Intake and Energy Availability

The results of the estimated EI and energy expenditure estimates of the participants are shown in Table 3. The mean intake of the sample of climbers was 1803.70 kcal-d1 (SD: 554.18), with a mean EA of 33.01 kcal-kg FFM1-d1 (SD: 9.02). Of the 45 subjects, only 8.9% (13.6% and 4.3% of males and females, respectively) had an EA > 45 kcal-kg FFM1-d1. Some (55.6%) of the athletes (54.5% and 56.5% of males and females, respectively) had suboptimal EA values of between 30 and 45 kcal-kg FFM^−1^d^−1^; while 35.6% (31.8% and 39.1% of males and females, respectively) of the 45 athletes were classified as having LEA (<30 kcal-kg FFM^−1^d^−1^).

Although statistically significant sex differences with a strong effect size were observed in the total daily energy intake (*p* < 0.010, d = 1.747, CI = 1.049–2.430), no statistically significant differences were observed between men and women in the EEE as well as in the energy intake and energy availability of athletes when these values were expressed in relation to fat-free mass.

Statistically significant (*p* < 0.050) medium correlations were also observed between daily energy intake (kcal/day) and the endomorph (r = −0.346) and mesomorph (r = 0.406) somatotypes (Table 4). A positive and negative association of fat-free mass and fat mass, respectively, with appetite in athletes may be behind a higher or lower intake in these athletes [48].

#### 3.3.2. Macronutrient Intake

The results of macronutrient intake are shown in Table 3. Regarding CHO intake, 57.8% of the athletes (50% male and 65.2% female) reported intakes below the minimum value of 3 g-kg BM^−1^ recommended for these athletes, while the remaining 42.2% were within the recommendations of 3–7 g CHOs-kg BM^−1^ per day.

Regarding protein intake, most of the 45 athletes (51.1% of the total; 50% and 52.2% of men and women, respectively) reported intakes in relation to body mass within the recommended values for each discipline (1.4–2 g-kg BM^−1^ in boulderers and 1.2–1.8 g-kg BM^−1^ for the rest), while 31.1% (22.7% men and 39.1% women) and 17.8% (27.3% men and 8.7% women) presented intakes below and above these recommendations, respectively.

In terms of fat intake, no climber reported fat intakes below 20% of the total energy intake, with 82.2% of athletes (77.3% male and 87% female) reporting an energy intake from fat intake greater than 35% of the total energy intake.

Statistically significant differences were observed between males and females with a strong effect size for the total daily intake of carbohydrates (*p* < 0.010, d = 1.245, and CI = 0.599–1.880), proteins (*p* < 0.010, d = 1.788, and CI = 1.085–2.880), and fats (*p* < 0.010, d = 1.485, and CI = 0.815–2.141), but no such differences were observed when expressed in relation to the athletes’ body masses or as a percentage contribution to total energy intake. Statistically significant gender differences in another parameter were only observed when protein intake was related to athlete body mass (*p* < 0.050).

Moderate significant correlations (*p* < 0.050) were found between carbohydrate intake and the mesomorph component of the athletes (r = 0.371), as well as in the endomorph and mesomorph somatotypes for protein intake (r = −0.451 and r = 0.380 for endomorph and mesomorph, respectively) and fat (r = −0.303 and r = 0.382 for endomorph and mesomorph, respectively) (Table 4).

#### 3.3.3. Micronutrient Intake

The results of the intake of different micronutrients are shown in Table 5 and Table 6. Within the vitamins, the variables with the highest prevalence of subjects with intakes below the Spanish RDA were as follows: vitamin E with 66.7% of the athletes (59.1% of men and 73.9% of women), folic acid with 53.3% (59.1% men and 47.8% women), thiamine with 42.2% (31.8% men and 52.2% women), vitamin D with 42.2% (36.4% men and 47.8% women), riboflavin with 40% (36.4% men and 43.5% women), and vitamin A with 35.6% (40.9% men and 30.4% women). Others, such as vitamin B6 (20%), vitamin C (17.8%), niacin (15.6%), and vitamin B12 (6.7%), showed the lowest prevalence values of climbers with deficient vitamin intakes.

On the other hand, regarding mineral intake, of the 45 athletes, 88.9% (81.8% of men and 95.7% of women), 64.4% (40.9% men and 87% women), 57.8% (50% men and 65.2% women), 51.1% (40.9% men and 60.9% women), and 51.1% (27.3% men and 73.9% women) reported intakes of iodine, potassium, calcium, magnesium, and selenium, respectively, below the recommended daily intakes for the Spanish population. However, 48.9% (27.3% men and 69.6% women), 46.7% (9.1% men and 82.6% women), and 44.4% (45.5% men and 43.5% women) of climbers reported deficient intakes of sodium, iron, and zinc, respectively. Only 13% of female athletes reported phosphorus intakes below the RDA, with 100% of male athletes having adequate intakes of this mineral.

Statistically significant differences were observed in the daily intakes of thiamine (*p* = 0.011, d = 0.560, and CI = −0.040–1.153), vitamin B6 (*p* = 0.046, d = 0.613, and CI = 0.011–1.208), phosphorus (*p* < 0.010, d = 1.273, and CI = 0.623–1.910), potassium (*p* < 0.010, η^2^ = 1.113, and CI = 0.478–1.910), magnesium (*p* = 0.013, d = 0.775, and CI = 0.163–1.377), iron (*p* = 0.031, d = 0.568, and CI = −0.0332–1.162), zinc (*p* < 0.010, d = 0.117, and CI = −0.468–0.702), iodine (*p* = 0.011, d = 0.533, and CI = −0.065–1.125) and selenium (*p* < 0.010, d = 1.244, and CI = 0.597–1.878) between the male and female athletes.

Statistically significant correlations (*p* < 0.050) were observed between daily niacin intake and IRCRA score (r = −0.420); between endomorphy values and daily intakes of thiamine (r = −0.298), niacin (r = −0.297), phosphorus (r = −0.300), zinc (r = −0.313), iodine (r = −0.410), and selenium (r = −0.348); and between mesomorphy values and intakes of phosphorus (r = 0.321), potassium (r = 0.339), zinc (r = 0.333), and selenium (r = 0.441) (Table 6).

#### 3.3.4. Supplement Use

A total of 37.8% of participants (40.9% of males and 34.8% of females) reported taking some type of supplement during the study period. The most commonly used supplements were protein powder (*n* = 7) and creatine (*n* = 7). Vitamin B12 was the third most used supplement by climbers (*n* = 5), followed by vitamin C and vitamin B6 (*n* = 3). Other reported supplements (n ≤ 2) were vitamin D, iron, riboflavin, omega-3, magnesium, essential amino acids, collagen, inositol, caffeine, folic acid, vitamin E, zinc, and calcium.

Athletes who supplemented protein (*p* < 0.010; d = 1.183; and CI = 0.333–2.020), vitamin D (*p* = 0.018; d = 8.707; and CI = 6.369–11.005), riboflavin (*p* = 0.024; d = 2.513; and CI = 0.987 -4.014), vitamin B6 (*p* < 0.010; d= 1.688; and CI = 0.455 -2.904), vitamin B12 (*p* < 0.010; d = 4,634; and CI = 3.273–5.969), iron (*p* = 0.024; d = 2.805; and CI = 1.255–4.327), and magnesium (*p* <= 0.024; d = 2.529; CI = 1.001–4.030) showed significantly different intakes of the respective nutrients with strong effect sizes relative to athletes who did not take such supplements.

## 4. Discussion

To date and to the knowledge of this research group, this is the first study to assess energy availability in Spanish climbing athletes. Performing a joint dietary and anthropometric assessment by subgroups of levels and genders allows for a complete perspective to be obtained on the nutritional situations and the differential aspects between athletes at the dietary and body levels. A nutritional study also allows us to assess part of the health and sporting statuses of these athletes [20], offering areas for improvement in their optimisation.

The findings of the present study show a generalised nutritional inadequacy in these athletes with respect to the recommended values, with a close link between the effect of the sporting practise of climbing on the body composition and the dietary habits of these athletes. These facts highlight the need for greater nutritional attention in this group of athletes in search of optimising their performance and health.

### 4.1. Body Composition

The first phase of this study focused on the analysis of the body compositions and anthropometric variables of the athletes and their relationship with the athletes’ skill levels. In the present study, statistically significant negative correlations were observed for all variables linked to the fat masses of the athletes, with particularly marked differences being found in the male athletes. These findings are fully in line with the results obtained in previous studies, where body fat levels are shown to be a differential aspect among the most skilled climbers with respect to other athletic groups or controls [12,49]. This tendency towards a reduced level of body fat in these athletes may be associated with other determinants of climbing performance, such as finger strength or upper body power [10,50,51].

However, although these facts serve to highlight once again the role of the endomorph component as a determinant of success in this sporting discipline [52], it is possible that this influence may be partially increased in male athletes. Even so, taking into account the relatively low levels of body fat shown by these athletes [33,35], adequate monitoring of body composition by the athlete’s environment, especially in more skilled climbers with a greater involvement in the daily practise of climbing, may be a prudent strategy in the prevention of health problems and the deterioration of sporting performance [15]. More studies are needed to examine the effects of relatively low levels of body fat on various parameters of health and sports performance in climbing athletes. On the other hand, previous studies have similarly shown that greater climbing ability is associated with greater forearm circumference [53]. Given the relationship of this anthropometric variable with maximal grip strength [54] and the musculature involved in climbing [53], it may be of interest to consider forearm circumference in the daily practise of the sport climbing athlete for the assessment of athletic performance improvement [55].

### 4.2. Nutritional Intake

The second part of this study focused on the assessment of dietary intake by athletes and the study of differences and associations between gender and climbing level, respectively.

Firstly, a high prevalence of athletes with compromised energy availability states was observed. These results highlight the concern observed in previous studies in climbers internationally, where a high number of climbing athletes had LEA states [42,44,56]. Although the energy intakes of the Spanish athletes did not show notable differences from those obtained in previous studies in Polish and Scottish climbing athletes [42,57], it is important to interpret the results obtained while cautiously considering the limitations intrinsic to the use of self-reporting tools and the use of metabolic equivalents (METs) during the estimation of athletes’ energy availability [58]. Even so, it is important to consider the relevance of these results and the need for further optimisation of the energy statuses of sport climbing athletes [13], especially because of the implications that a poor energy status has on the health status and sport performance of athletes [59]. Studies assessing the chronic effects of a state of LEA on the health status and athletic performance of these athletes are needed.

In relation to the intake of macronutrients, a generalised intake of carbohydrates by Spanish athletes below the recommendations for the sport in question stands out, with these values being even lower than those obtained in previous research on climbing athletes [21,42,44,56,58,60]. Deficient carbohydrate intakes could lead to a reduction in body glycogen levels and accelerate the onset of fatigue and the deterioration of sports performance in these athletes [61,62].

In contrast, and in agreement with much of the literature [21,44,56,60], the reduction in carbohydrate intake contrasted with a large number of athletes with fat intakes in relation to total energy intake above the recommendations of 35%. Considering the effects of low-carbohydrate and high-fat diets on exercise economy [63] and the importance of this factor in the performance of climbing athletes [5], it seems particularly interesting to try to recommend an increase in energy intake and improve energy availability status by optimising carbohydrate intake in these athletes. It is speculative that this strategy may be of relevance to climbers who perform outdoor climbing in prolonged or successive sessions where lactate levels may be increased [64].

Although the protein intake of Spanish athletes did not differ particularly from the values found in other climbing athletes [21,44,58], several athletes showed intakes below the recommendations. Considering the large energy deficit status of these athletes and the high frequency of isometric contractions performed during climbing [13,65], it may be especially important to take care of protein intake in climbing athletes in view of maintaining maximal levels of fat-free mass and grip strength [66,67]. However, focusing on energy intake in relation to increases in nutritional carbohydrate intake seems to be the most sensible recommendation based on the findings of the present study.

A large proportion of the athletes showed intakes below the recommendations for most of the nutrients analysed, similar to the findings available from previous studies in climbers [21,42,44,56,60]. However, these data should be interpreted with caution, given the need for longer dietary recording times to estimate individual intake more accurately [68,69]. Also, the use of population-based reference values such as RDAs or the high use of dietary supplements by climbing athletes may, respectively, aggravate or mask the diagnosis of specific nutritional deficiencies [70,71,72,73].

However, these data raise concerns about the statuses of certain vitamins and minerals, especially micronutrients that may compromise athletic performance in these sports disciplines to a greater extent, such as iron, vitamin D, calcium, B vitamins, magnesium, and zinc [13,14]. The consideration of monitoring the dietary intake of these micronutrients in conjunction with biochemical and even body composition assessments [74] may be a prudent strategy in this group of athletes before considering the use of any dietary supplementation [68].

However, it can be deduced that these problems and deficient micronutrient intakes can be reversed by adjusting the energy intake of athletes [59,75]. Therefore, improving the nutritional status and energy status of climbers by adapting their dietary habits to the specific requirements of their sporting discipline seems to be the priority strategy in these athletes as in other Olympic sporting disciplines [76,77,78], and the safe, effective, and necessary use of certain nutritional supplements should be considered for situations involving diagnosed deficiencies, athletes who follow restrictive dietary patterns, or where there is a potential effect on the athlete’s sporting performance [20,73,79,80,81,82]. However, more studies are needed to monitor the statuses of different micronutrients in the climbing athlete, as well as their health and sporting effects.

## 5. Limitations

To the knowledge of the present research group, this is one of the first studies to establish a relationship between nutritional intake and energy availability in sport climbing athletes with their respective anthropometric and body variables. These findings may encourage the development of future research that considers the implications of nutrition on sport performance and health in this population group. In addition, the inclusion of previously validated nutritional and body assessment methods in the present study facilitates the comparability of the results with those obtained in external research.

However, this study is not without limitations.

The main limiting factor of the present study is the bias arising from the use of self-reporting tools, questionnaires, or records, and there may be a risk of underestimation of the intakes and counts obtained [83]. Also, the short duration of dietary diaries may be a limiting aspect in accurately estimating nutritional intakes at the individual level, especially regarding micronutrients [68,69]. A biochemical assessment of sport climbing athletes could be an interesting addition to future research.

Finally, the variability and method-specific biases present in measurement techniques such as anthropometry could be added biases when analysing and interpreting the different data obtained [84,85]. The use of validated reference methods may be advisable for body composition assessments in climbing athletes in future studies.

## 6. Conclusions

A generalised nutritional inadequacy was observed in Spanish climbing athletes with respect to the specific recommendations for this sport discipline. Sport climbing athletes presented a mean energy availability value of 33.01 kcal-kg FFM^−1^d^−1^ (SD: 9.02), with only 8.9% of the values being above 45 kcal-kg FFM^−1^d^−1^, represented largely by insufficient carbohydrate intakes. Statistically significant negative correlations were observed between the athletes’ ability levels and all variables related to athlete fat mass (*p* < 0.010).

These findings highlight the need for an adequate nutritional intervention and an optimisation of the energy intake of the sport climbing athlete according to the specific demands derived from their sport practise. Likewise, the implementation of nutritional monitoring in daily practise for the sport climbing athlete may be an interesting strategy in the evaluation of the athlete’s health and sporting status.

Future research evaluating the effects of an inadequate nutritional status and prolonged LEA situations on the health and sporting performance of these athletes is necessary.

## Figures and Tables

**Table 1:** Demographic and sporting characteristics of participants.

		Total (*n* = 46)		Males (*n* = 22)		Females (*n* = 24)		*p*	Effect Size **	
		Mean	SD	Mean	SD	Mean	SD		*d*	IC (95%)
Age (years)		30	9	32	10	28	8	0.190 #	0.393	(−0.194–0.975)
Weight (kg)		59.56	10.12	67.78	7.77	52.03	4.65	<0.001 *	2.487	(1.703–3.256)
Height (cm)		167.73	8.62	174.49	6.89	161.53	4.27	<0.001 #	2.284	(1.527–3.026)
BMI (kg/m^2^)		21.02	1.96	22.20	1.71	19.93	1.52	<0.001 #	1.409	(0.754–2.051)
IRCRA score		19.07	4.72	20.00	4.61	18.21	4.75	0.189 *	0.383	(−0.203–0.965)
Experience (years)		7.66	6.63	8.57	7.17	6.83	6.13	0.460 *	0.261	(−0.321–0.841)
Volume of climbing per week (hours)		2.71	1.69	2.70	1.73	2.73	1.70	0.921 *	−0.018	(−0.596–0.561)
Time spent climbing in the past 3 months (%)	Boulder	53.91	28.26	55.68	30.37	52.29	26.74	0.689 #	0.119	(−0.461–0.697)
	Speed	1.54	10.32	3.18	14.92	0.04	0.20	0.926 *	0.305	(−0.279–0.885)
	Lead	44.54	28.29	41.14	30.12	47.67	26.77	0.440 #	−0.230	(−0.809–0.352)
Time spent climbing in the past 12 months (%)	Boulder	53.80	25.41	53.64	30.01	53.96	21.01	0.966 #	−0.013	(−0.591–0.566)
	Speed	0.89	5.90	1.82	8.53	0.04	0.20	0.926 *	0.301	(−0.282–0.882)
	Lead	42.48	24.57	40.00	28.45	44.75	20.75	0.519 #	−0.192	(−0.771–0.389)
Time spent climbing in the past 3 months (%)	Inside	71.52	23.71	74.09	21.02	69.17	26.15	0.594 *	0.207	(−0.375–0.785)
	Outside	28.48	23.71	25.91	21.02	30.83	26.15	0.594 *	−0.207	(−0.785–0.375)
Time spent climbing in the past 12 months (%)	Inside	67.61	23.45	66.36	24.36	68.75	23.04	0.894 *	−0.101	(−0.679–0.479)
	Outside	30.22	21.60	29.09	20.39	31.25	23.04	0.722 *	−0.099	(−0.677–0.480)

SD: standard deviation. * Mann–Whitney U test; ** Cohen’s d index; # independent samples *t*-test.

**Table 2:** Variables related to body composition and correlations with IRCRA scale.

	Males (*n* = 22)						Females (*n* = 24)						IRCRA Scale	*p*
	Intermediate (Level 2) (*n* = 7)		Advanced (Level 3) (*n* = 8)		Elite and High Elite (Levels 4 and 5) (*n* = 7)		Intermediate (Level 2) (*n* = 7)		Advanced (Level 3) (*n* = 10)		Elite and High Elite (Levels 4 and 5) (*n* = 7)			
	Mean	SD	Mean	SD	Mean	SD	Mean	SD	Mean	SD	Mean	SD	Spearman’s R	
Weight (kg)	69.77	5.62	66.35	5.74	67.41	11.56	54.94	5.40	51.12	3.33	50.40	4.78	0.012	0.463 #
Height (cm)	172.69	3.21	173.50	5.01	177.43	10.55	162.09	6.74	162.32	2.39	159.86	3.34	0.188	0.929 *
BMI (kg/$m^{2})$	23.38	1.50	22.03	1.51	21.22	1.58	20.91	1.52	19.40	1.18	19.72	1.69	−0.159	0.032 *
Sitting height (cm)	88.01	3.53	91.10	2.46	92.66	7.44	86.39	2.56	85.84	2.65	84.93	1.21	0.172	0.606 *
Arm span (cm)	176.71	6.99	177.55	5.84	182.21	10.35	164.33	6.83	162.92	3.41	163.74	5.57	0.147	0.737 #
∑6 skinfolds (mm)	61.52	16.41	47.25	12.22	34.92	3.05	66.31	6.10	64.09	8.62	58.70	12.65	***−0.573***	0.007 *
∑8 skinfolds (mm)	79.54	21.53	58.91	15.70	44.19	3.68	80.16	8.07	77.08	10.17	69.28	15.34	***−0.616***	0.002 *
Arm relaxed girth (cm)	30.83	1.32	29.89	1.86	28.56	2.49	24.89	1.91	25.30	1.15	25.09	1.48	−0.018	0.458 #
Arm flexed girth (cm)	33.32	1.17	32.92	1.78	32.30	3.43	25.98	1.76	26.58	1.24	27.16	1.41	0.204	0.989 #
Forearm girth (cm)	26.91	3.77	27.84	1.50	27.70	1.97	22.84	0.57	23.08	1.14	23.42	0.72	**0.342**	0.586 #
Thigh middle girth (cm)	50.12	2.24	48.16	2.77	47.06	3.74	47.26	2.55	43.68	2.72	44.34	3.11	−0.150	0.026 *
Calf girth (cm)	36.40	2.10	36.18	1.53	35.78	2.47	34.06	1.57	32.76	2.36	32.83	1.34	0.002	0.495 *
Hand length (cm)	18.54	0.92	18.39	0.57	18.57	1.24	16.34	0.86	17.07	0.56	16.81	0.68	0.183	0.871 *
Humerus breadth (cm)	7.61	0.20	7.57	0.38	7.67	0.34	6.13	0.37	6.43	0.27	6.43	0.37	0.247	0.746 #
Bi-styloid breadth (cm)	5.89	0.38	5.85	0.26	6.11	0.44	5.07	0.14	5.21	0.20	5.20	0.16	0.281	0.642 #
Femur breadth (cm)	9.83	0.26	9.95	0.39	10.17	0.53	8.97	0.24	8.84	0.29	8.77	0.35	0.164	0.851 *
Fat mass (kg)	12.18	2.86	9.66	2.94	6.92	1.91	12.75	2.29	11.66	1.42	10.00	2.52	***−0.590***	<0.001 *
Bone mass (kg)	11.94	0.66	12.09	0.93	13.14	1.88	9.21	0.73	9.30	0.43	9.04	0.43	0.191	0.900 #
Muscle mass (kg)	31.18	2.54	30.70	2.51	30.37	5.33	20.57	1.33	20.19	1.84	20.10	1.52	0.163	0.903 #
% fat mass	17.35	3.47	14.52	4.05	10.25	1.88	23.09	2.43	22.81	2.46	19.77	4.26	***−0.497***	0.055 #
Endomorphy	2.99	1.01	2.08	0.57	1.46	0.19	2.99	0.38	2.89	0.49	2.55	0.79	***−0.589***	0.003 *
Mesomorphy	6.19	0.70	6.04	0.94	5.61	0.58	3.87	1.20	3.94	0.70	4.35	0.66	0.143	0.939 *
Ectomorphy	2.16	0.67	2.82	0.85	3.46	0.43	2.66	0.95	3.46	0.66	3.14	0.92	**0.308**	0.008 *

SD: standard deviation; bolded text: <0.050; bolded + italicized text: <0.010; * one-factor ANOVA; # Kruskal–Wallis H-test.

**Table 3:** Energy requirements, EA, and macronutrient intake.

		Total (*n* = 45)		Males (*n* = 22)		Females (*n* = 23)		*p*	Effect Size **	
		Mean	SD	Mean	SD	Mean	SD		*d*	IC (95%)
Energy Requirements										
EEE (kcal/day)		184.33	126.64	211.72	152.29	159.21	93.95	0.235 *	0.419	(−0.168–1.002)
Energy Intake										
Total kcal/day		1803.70	554.18	2176.98	505.10	1446.64	313.17	<0.001 #	1.747	(1.049–2.430)
Total kcal·kg·FFM^−1^d^−1^		36.82	8.44	37.60	8.23	36.07	8.76	0.551	0.179	(−0.407–0.764)
Energy Availability (kcal·kg FFM^−1^d^−1^)		33.01	9.02	34.06	8.63	32.01	9.45	0.451 #	0.227	(−0.361–0.812)
Carbohydrate Intake										
Total g/day		172.01	71.07	210.60	73.75	135.09	44.66	<0.001 *	1.245	(0.599–1.880)
g/kg·day		2.87	1.02	3.12	1.07	2.63	0.92	0.105 #	0.493	(−0.103–1.084)
% Total Energy Intake		37.31	7.07	38.04	6.93	36.62	7.28	0.504 #	0.201	(−0.386–0.786)
Protein Intake										
Total g/day		93.89	31.06	115.08	27.29	73.62	18.43	<0.001 #	1.788	(1.085–2.476)
g/kg·day		1.57	0.43	1.71	0.44	1.43	0.38	0.021 *	0.703	(0.096–1.302)
% Total Energy Intake		21.11	4.05	21.50	3.89	20.73	4.25	0.527 #	0.190	(−0.397–0.775)
Fat Intake										
Total g/day		80.84	25.49	96.42	24.58	65.93	15.73	<0.001 #	1.485	(0.815–2.141)
% Total Energy Intake	Total	40.77	6.10	40.14	5.94	41.38	6.31	0.504 #	−0.201	(−0.786–0.386)
	Saturated Fatty Acids	10.65	2.27	10.52	1.97	10.77	2.56	0.714 #	−0.110	(−0.694–0.476)
	Monounsaturated Fatty Acids	16.95	4.11	17.52	4.43	16.40	3.80	0.366 #	0.273	(−0.316–0.858)
	Polyunsaturated Fatty Acids	7.36	2.92	7.26	2.53	7.46	3.31	0.964 *	−0.066	(−0.650–0.519)

SD: standard deviation. * Mann–Whitney U test; ** Cohen’s d index; # independent samples *t*-test.

**Table 4:** Correlations between dietary variables, somatotype and ability, and sport experience level.

	IRCRA Score	Experience (Years)	Endomorphy	Mesomorphy	Ectomorphy
	R	R	R	R	R
Energy intake (kcal/day)	0.900 *	0.033 *	**−0.346** #	***0.406*** #	0.018 #
EA (kcal·kg·FFM^−1^d^−1^)	−0.245 *	−0.170 *	0.061 #	−0.136 #	0.256 #
Carbohydrate (g/day)	−0.038 *	−0.044 *	−0.199 *	**0.371** *	−0.039 *
Fat (g/day)	0.185 *	0.118 *	**−0.303** #	**0.382** #	0.044 #
Protein (g/day)	0.236 *	−0.021 *	***−0.451*** #	**0.380** #	0.090 #
Thiamine (mg/day)	0.113 *	0.076 *	**−0.298** *	0.229 *	0.066 *
Riboflavin (mg/day)	0.120 *	−0.126 *	−0.241 *	0.187 *	0.093 *
Niacin (mg/day)	***0.420*** *	0.124 *	**−0.297** *	0.027 *	0.189 *
Vitamin B6 (mg/day)	0.179 *	−0.049 *	−0.213 #	0.132 #	0.128 #
Folic acid (µg/day)	0.090 *	−0.107 *	−0.022 *	−0.005 *	0.165 *
Vitamin B12 (µg/day)	0.058 *	−0.064 *	−0.141 *	0.206 *	−0.027 *
Vitamin C (mg/day)	−0.041 *	−0.198 *	0.041 *	−0.045 *	0.093 *
Vitamin A (µg/day)	0.023 *	0.067 *	0.161 *	−0.112 *	0.015 *
Vitamin D (µg/day)	0.042 *	−0.026 *	0.107 *	0.127 *	−0.133 *
Vitamin E (mg/day)	0.093 *	0.183 *	−0.087 *	0.167 *	0.075 *
Calcium (mg/day)	−0.093 *	−0.129 *	0.014 #	0.141 #	0.054 #
Phosphorus (mg/day)	0.119 *	−0.078 *	**−0.300** #	**0.321** #	0.039 #
Potassium (mg/day)	0.118 *	0.009 *	−0.258 *	**0.339** *	−0.014 *
Magnesium (mg/day)	−0.051 *	−0.096 *	−0.041 *	0.219 *	0.024 *
Iron (mg/day)	0.062 *	−0.050 *	−0.251 *	0.217 *	0.038 *
Zinc (mg/day)	0.173 *	−0.107 *	**−0.313** *	**0.333** *	0.066 *
Iodine (µg/day)	0.207 *	0.076 *	***−0.410*** *	0.185 *	0.182 *
Selenium (µg/day)	0.071 *	−0.050 *	**−0.348** *	***0.441*** *	−0.151 *

# Pearson’s R; * Spearman’s R; bolded letter: <0.050; bolded and italicized letter: <0.010.

**Table 5:** Intake of vitamins and adjustment to Spanish RDAs.

		Total (*n* = 45)		Males (*n* = 22)		Females (*n* = 23)		*p*	Effect Size **	
		Mean	SD	Mean	SD	Mean	SD		*d*	IC (95%)
Thiamine	Total mg/day	1.35	0.62	1.53	0.51	1.19	0.69	0.011 *	0.560	(−0.040–1.153)
	% RDA	122.94	56.76	127.23	42.16	118.83	68.63	0.153 *	0.147	(−0.439–0.718)
Riboflavin	Total mg/day	1.85	1.11	2.05	1.14	1.65	1.07	0.054 *	0.365	(−0.227–0.952)
	% RDA	129.82	78.25	129.34	71.26	130.27	86.00	0.555 *	−0.012	(−0.596–0.573)
Niacin	Total mg/day	75.32	124.32	33.89	15.02	114.95	165.34	0.256 *	−0.683	(−1.281–−0.077)
	% RDA	513.90	895.30	192.78	83.26	821.06	1181.00	0.683 *	−0.742	(−1.343–−0.133)
Vitamin B6	Total mg/day	2.13	0.89	2.40	0.78	1.88	0.93	0.046 #	0.613	(0.011–1.208)
	% RDA	157.19	63.77	161.49	51.63	153.09	74.51	0.664 #	0.131	(−0.455–0.715)
Folic acid	Total µg/day	321.96	143.32	333.22	129.91	311.19	157.24	0.525 *	0.152	(−0.434–0.737)
	% RDA	107.32	47.77	111.07	43.30	103.73	52.41	0.525 *	0.152	(−0.434–0.737)
Vitamin B12	Total µg/day	27.39	72.29	20.49	60.13	34.00	83.10	0.200 *	−0.186	(−0.770–0.401)
	% RDA	1369.51	3614.28	1024.25	3006.56	1699.76	4155.03	0.200 *	−0.186	(−0.770–0.401)
Vitamin C	Total mg/day	168.44	188.04	146.13	101.18	189.77	244.89	0.856 *	−0.231	(−0.816–0.357)
	% RDA	280.73	313.40	243.55	168.63	316.29	408.16	0.856 *	−0.231	(−0.816–0.357)
Vitamin A	Total µg/day	874.42	570.67	868.02	714.27	880.55	405.28	0.329 *	−0.022	(−0.606–0.563)
	% RDA	135.03	86.30	122.77	102.56	146.76	67.55	0.073 *	−0.277	(−0.863–0.312)
Vitamin D	Total µg/day	10.33	18.88	12.78	24.87	7.99	10.52	0.555 *	0.253	(−0.335–0.838)
	% RDA	206.69	377.52	255.67	497.32	159.84	210.40	0.555 *	0.253	(−0.335–0.838)
Vitamin E	Total mg/day	19.28	38.85	15.23	6.06	23.16	54.32	0.107 *	−0.203	(−0.788–0.384)
	% RDA	128.54	258.97	101.52	40.39	154.39	362.15	0.107 *	−0.203	(−0.788–0.384)

SD: standard deviation. * Mann–Whitney U test; ** Cohen’s d index; # independent samples *t*-test.

**Table 6:** Mineral intake and adjustment to Spanish RDA.

		Total (*n* = 45)		Males (*n* = 22)		Females (*n* = 23)		*p*	Effect Size **	
		Mean	SD	Mean	SD	Mean	SD		*d*	IC (95%)
Calcium	Total mg/day	866.88	355.37	949.10	371.21	788.24	328.35	0.131 #	0.460	(−0.135–1.050)
	% RDA	96.25	39.61	105.46	41.25	87.45	36.71	0.129 #	0.462	(−0.133–1.052)
Phosphorus	Total mg/day	1226.44	413.06	1454.23	392.27	1008.55	304.69	<0.001 #	1.273	(0.623–1.910)
	% RDA	171.41	59.57	204.66	58.01	139.60	41.50	<0.001 #	1.295	(0.644–1.934)
Potassium	Total mg/day	2897.10	1198.47	3496.63	1253.12	2323.63	818.83	0.001 *	1.113	(0.478–1.738)
	% RDA	93.45	38.66	112.79	40.42	74.96	26.41	0.001 *	1.113	(0.478–1.738)
Magnesium	Total mg/day	349.70	171.95	413.72	192.97	288.46	124.79	0.013 *	0.775	(0.163–1.377)
	% RDA	106.93	49.40	118.21	55.13	96.15	41.60	0.188 *	0.453	(−0.142–1.043)
Iron	Total mg/day	14.60	6.15	16.33	5.36	12.94	6.51	0.031 *	0.568	(−0.032–1.162)
	% RDA	126.12	73.12	177.91	62.23	76.57	41.80	<0.001 *	1.920	(1.202–2.623)
Zinc	Total mg/day	10.41	9.36	10.98	3.02	9.87	12.88	<0.001 *	0.117	(−0.468–0.702)
	% RDA	125.89	132.84	113.55	32.39	137.69	184.37	0.296 *	−0.180	(−0.765–0.406)
Iodine	Total µg/day	97.41	114.14	127.76	151.42	68.38	48.66	0.011 *	0.533	(−0.065–1.125)
	% RDA	64.94	76.09	85.17	100.95	45.59	32.44	0.011 *	0.533	(−0.065–1.125)
Selenium	Total µg/day	61.47	41.33	83.90	47.71	40.02	16.12	<0.001 *	1.244	(0.597–1.878)
	% RDA	114.03	74.52	154.16	86.23	75.64	29.30	<0.001 *	1.231	(0.585–1.864)

SD: standard deviation. * Mann–Whitney U test; ** Cohen’s d index; # independent samples *t*-test.

## Data Availability

There are restrictions on the availability of data for this trial due to the signed consent agreements around data sharing, which only allow access to external researchers for studies following the project’s purposes. Requestors wishing to access the trial data used in this study can make a request to mariscal@ugr.es.
